# Supplementary material for: Empirical methods for controlling false positives and estimating confidence in ChIP-Seq peaks
Source: BMC Bioinformatics. 2008 Dec 5;9:523. doi: 10.1186/1471-2105-9-523 (PMC2628906; doi:10.1186/1471-2105-9-523)
Supplement: Additional File 1 — A variety of html documents from the USeq web site detailing the available applications, their best usage, output file type descriptions, command line menus, etc. [file 1471-2105-9-523-S1.zip › USeqUserGuides/JavaDoc.zip!/JavaDoc/util/bio/wrappers/BlastResult.html]

BlastResult


|  |  |  |
| --- | --- | --- |
| **Overview** \| **Package** \| **Class** \| **Use** \| **Tree** \| **Deprecated** \| **Index** \| **Help** | |  |
| **PREV CLASS**   **NEXT CLASS** | **FRAMES**    **NO FRAMES**     **All Classes** |  |
| SUMMARY: NESTED \| FIELD \| CONSTR \| METHOD | DETAIL: FIELD \| CONSTR \| METHOD |  |


---


## util.bio.wrappers Class BlastResult

```
java.lang.Object
  util.bio.wrappers.BlastResult
```

---

``` public class BlastResult extends java.lang.Object ```

Information related to a blast results.

---

**Constructor Summary**

`BlastResult()`


| **Method Summary** | |
| --- | --- |
| `java.lang.String[]` | `getAlignment()` |
| `java.lang.String` | `getExpect()` |
| `java.lang.String` | `getGaps()` |
| `java.lang.String` | `getIdentities()` |
| `java.lang.String` | `getMatchId()` |
| `java.lang.String` | `getMatchLength()` |
| `java.lang.String` | `getMatchName()` |
| `java.lang.String` | `getScore()` |
| `java.lang.String` | `getStrand()` |
| `void` | `setAlignment(java.lang.String[] alignment)` |
| `void` | `setExpect(java.lang.String expect)` |
| `void` | `setGaps(java.lang.String gaps)` |
| `void` | `setIdentities(java.lang.String identities)` |
| `void` | `setMatchId(java.lang.String matchId)` |
| `void` | `setMatchLength(java.lang.String matchLength)` |
| `void` | `setMatchName(java.lang.String matchName)` |
| `void` | `setScore(java.lang.String score)` |
| `void` | `setStrand(java.lang.String strand)` |
| `java.lang.String` | `toString()` |

**Methods inherited from class java.lang.Object**

`equals, getClass, hashCode, notify, notifyAll, wait, wait, wait`

**Constructor Detail**

### BlastResult

```
public BlastResult()
```


**Method Detail**

### toString

```
public java.lang.String toString()
```

:   **Overrides:**: `toString` in class `java.lang.Object`

---


### getAlignment

```
public java.lang.String[] getAlignment()
```

---


### setAlignment

```
public void setAlignment(java.lang.String[] alignment)
```

---


### getExpect

```
public java.lang.String getExpect()
```

---


### setExpect

```
public void setExpect(java.lang.String expect)
```

---


### getGaps

```
public java.lang.String getGaps()
```

---


### setGaps

```
public void setGaps(java.lang.String gaps)
```

---


### getIdentities

```
public java.lang.String getIdentities()
```

---


### setIdentities

```
public void setIdentities(java.lang.String identities)
```

---


### getMatchLength

```
public java.lang.String getMatchLength()
```

---


### setMatchLength

```
public void setMatchLength(java.lang.String matchLength)
```

---


### getMatchId

```
public java.lang.String getMatchId()
```

---


### setMatchId

```
public void setMatchId(java.lang.String matchId)
```

---


### getScore

```
public java.lang.String getScore()
```

---


### setScore

```
public void setScore(java.lang.String score)
```

---


### getStrand

```
public java.lang.String getStrand()
```

---


### setStrand

```
public void setStrand(java.lang.String strand)
```

---


### getMatchName

```
public java.lang.String getMatchName()
```

---


### setMatchName

```
public void setMatchName(java.lang.String matchName)
```


---


|  |  |  |
| --- | --- | --- |
| **Overview** \| **Package** \| **Class** \| **Use** \| **Tree** \| **Deprecated** \| **Index** \| **Help** | |  |
| **PREV CLASS**   **NEXT CLASS** | **FRAMES**    **NO FRAMES**     **All Classes** |  |
| SUMMARY: NESTED \| FIELD \| CONSTR \| METHOD | DETAIL: FIELD \| CONSTR \| METHOD |  |


---
